# Acute Febrile Illness and Influenza Disease Burden in a Rural Cohort Dedicated to Malaria in Senegal, 2012–2013

**DOI:** 10.1371/journal.pone.0143999

**Published:** 2015-12-17

**Authors:** Fatoumata Diene Sarr, Mbayame Niang, Diamilatou Thiam, Ndongo Dia, Abdoulaye Badiane, A. B. Ndao, Cheikh Sokhna, André Spiegel, Vincent Richard

**Affiliations:** 1 Epidemiology unit, Pasteur Institute in Dakar, Senegal; 2 Virological unit, Pasteur Institute in Dakar, Senegal; 3 Health district in Sokone, Ministry of Health, Sokone, Senegal; 4 Institut de recherche pour le développement, Dakar, Senegal; Public Health Agency of Canada, CANADA

## Abstract

**Background:**

African populations are considered to be particularly vulnerable to fever illnesses, including malaria, and acute respiratory disease, owing to limited resources and overcrowding. However, the overall burden of influenza in this context is poorly defined and incidence data for African countries are scarce. We therefore studied the fever syndrome incidence and more specifically influenza incidence in a cohort of inhabitants of Dielmo and Ndiop in Sokone district, Senegal.

**Methods:**

Daily febrile-illness data were prospectively obtained from January 2012 to December 2013 from the cohort of the villages of Dielmo and Ndiop, initially dedicated to the study of malaria. Nasopharyngeal swabs were collected from, and malaria diagnosis tests (thick blood smears) carried out on, every febrile individual during clinical visits; reverse transcriptase-polymerase chain reaction was used to identify influenza viruses in the samples. Binomial negative regression analysis was used to study the relationship between the monthly incidence rate and various covariates.

**Results:**

In Dielmo and Ndiop, the incidence of malaria has decreased, but fever syndromes remain frequent. Among the 1036 inhabitants included in the cohort, a total of 1,129 episodes of fever were reported. Influenza was present all year round with peaks in October-December 2012 and August 2013. The fever, ILI and influenza incidence density rates differed significantly between age groups. At both sites, the adjusted incidence relative risks for fever syndromes and ILI were significantly higher in the [6–24 months) than other age groups: 7.3 (95%CI: [5.7–9.3]) and 16.1 (95%CI: [11.1–23.3]) respectively. The adjusted incidence relative risk for influenza was significantly higher for the [0–6 months) than other age groups: 9.9 (95%CI: [2.9–33.6]). At both sites, incidence density rates were lowest among adults > = 50 years.

**Conclusions:**

In this rural setting in Senegal, influenza was most frequent among the youngest children. Preventive strategies targeting this population should be implemented.

## Introduction

African countries are burdened with a very heavy load of communicable diseases in addition to other severe health problems [1]. Clinically, influenza infection is not distinguishable from numerous other tropical infectious diseases involving fever. In the absence of laboratory confirmation, febrile illnesses are most often treated empirically as malaria. However, numerous studies show that influenza is prevalent in African regions [2,3]. In 2005, as a consequence of World Health Organization (WHO) advocacy, international resources were mobilized to help African countries respond to the threat of avian influenza. This involved the implementation and enhancement of influenza surveillance tools including the collection and analysis of virological and epidemiological data [3–11].

Every year, malaria and respiratory infections are among the leading causes of morbidity and mortality in Africa. In 2008, 99% of all deaths of children <5 years old associated with influenza infection occurred in developing countries [12]. The disease burden of influenza is undoubtedly far greater in Africa than in the developed world, owing to malnutrition and underlying tropical diseases which complicate the infection [13]. Influenza causes a significant burden of disease in countries throughout the tropics despite having historically been perceived as a mild or uncommon disease [3,14–17]. Data from tropical Asian countries show that rates of hospitalization and mortality specifically associated with influenza are similar to those in the United States [18,19]. In Sub-Saharan Africa, knowledge about the influenza burden is still limited [2], although work has started to address the issue of influenza in Africa [11].

Data about incidence and mortality are required as a basis for designing and implementing influenza control programs in Africa [20]. However, the gaps in the data for Africa are substantial, larger than in other tropical settings, and the data available about the burden of influenza are scarce [21]. Existing data, mostly from surveillance, are insufficient to provide meaningful estimates of influenza incidence. Prospective, multi-year, population-based data on laboratory-confirmed influenza infection taking into account malaria epidemiology are needed to describe the disease burden in African countries and provide information for prevention and control strategies during both inter-pandemic and pandemic periods [22].

Senegal is an equatorial country in West Africa with a mostly tropical climate; data are available from influenza surveillance in Dakar since 1996 thanks to a collaboration between the Ministry of Health and the Pasteur Institute in Dakar [4,8]. However, neither the disease burden nor the population at risk are well described. To rectify this situation, we tried to estimate the incidence of fever syndromes, influenza like illnesses and influenza infections in different age groups.

## Methods

### Study design

Between 1^st^ January 2012 and 31^th^ December 2013, we extended the prospective population-based study involving the inhabitants of the villages of Dielmo and Ndiop, Senegal: we implemented an additional longitudinal study of influenza, involving analysis of all febrile syndromes.

### Setting

The two villages, Dielmo and Ndiop, are located in a Sudan-savanna region of central Senegal, in the health district of Sokone. This rural setting has been used for extensive malaria studies [23].

### Participants

As part of the study designed in to investigate malaria, all episodes of fever among all the population of the two villages included in the cohort (n = 1036) were identified and recorded by fieldworkers.

### Follow-up

The cohort has been described elsewhere [23]. Each participant had an individual identification code for all the studies. On entry, socio economic information, the location of the residence in the villages and the size of the family were recorded. Field workers visited each household daily, 6 days/week (not on Sundays) to collect information about the presence or absence in the village of each cohort participant, their location when absent, and the presence of fever or other symptoms.

Free medical dispensaries were established in each of the two villages, and were open 24h/day, 7 days/week. All fever syndromes and fever-related symptoms identified by the fieldworkers were systematically examined, investigated and treated. Malaria testing (thick blood smears) was done by finger prick for all febrile cases. The study included systematic collection and testing of swabs from individuals with fever, not only from patients with influenza-like illness.

### Cases definition

In accordance with the sentinel surveillance system in Senegal [4] based on clinical pre-diagnostic data using standard WHO case definitions to ensure comparability, case definitions for the study were: fever (defined as an axillary temperature of more than 37.5° C), confirmed malaria cases (defined as fever and a positive result in a rapid diagnostic test), and influenza-like illness (defined as fever with cough or fever with sore throat).

### Virological analysis

Naso-pharyngeal and oral specimens were collected from all febrile patients included in the cohort in each village, put in 2 mL of viral transport medium (Universal Transport Medium, COPAN Diagnostics), and stored at 4°C before shipment to the NIC on a weekly basis. Samples were shipped at a controlled temperature (4°C) and processed immediately on arrival at the laboratory for virus detection, identification, and characterization by a real-time reverse-transcription polymerase chain reaction method (courtesy of the Centers for Disease Control [CDC], Atlanta).

### Statistical analysis

R software was used for statistical analysis [24]. We monitored the presence of each person in the village daily, and we measured the person-time incidence rates of febrile syndromes, influenza-like syndromes, malaria and influenza infection as a ratio: the number of fever episodes recorded during a given period divided by the number of person-days of follow-up included in the survey during the corresponding period. Mean annual incidence rates by period and by age group were calculated from the daily incidence rates on the basis of 365.25 days per year.

To take into account the overdispersion of the data, we used binomial negative regression to measure the relation between the incidence rate and the various covariates studied (age, gender, village.). All covariates with a p value lower than 0.20 were included in the multivariable model. Multivariable backward step-by-step binomial negative regressions were used to take into account confounders, bias, and interactions linked to the dependent variable. The [20–49 year) group was used as a reference group for comparisons between age group because of the lowest incidence density rates in this age group. We used logistic regression to estimate the relation between influenza infection and clinical symptoms. Statistical differences were considered significant for p-values <0.05.

### Ethical considerations

The project was approved by the Senegalese National Ethics committee of the Ministry of Health and the assembled village population. Audits were done in 2013 by the National Ethics Committee of Senegal. During the first step of the cohort study, all inhabitants were given full information about the study and provided written informed consent before inclusion. Respiratory specimens were collected for diagnosis only after written informed consent was granted, and recorded in a dedicated form, to local health-care workers by the patients or by the parents of minors. This process has been accepted by the national Ethics committee.

## Results

For the 1036 inhabitants included in the cohort, 529,279 follow-up days were reported: 275,068 in Dielmo and 254,211 in Ndiop (Table 1).

**Table 1:** Characteristics and incidence rates in the villages of Dielmo and Ndiop—January 2012–December 2013.

	Overall	Dielmo		Ndiop		
	N (%)	n	(%)	n	(%)	p-value
Nb of inhabitants	1,036	495	(48)	541	(52)	
Nb of follow-up days	529,279	275,068	(52)	254,211	(48)	
Nb of patients	543	271	(50)	272	(50)	
Sex						
Male	261 (48)	140	(52)	121	(44)	0.40*
Female	282 (52)	131	(48)	151	(56)	
Nb of fever episodes	1129	586	(52)	543	(48)	
Age						
Median	6.6	6.2		6.8		0.86^♭^
IQR	[12.1]	[11.9]		[12.5]		
Age groups						
[0–6months)	21 (2)	12	(2)	9	(2)	0.60*
[6–24months)	203 (18)	109	(19)	94	(17)	
[2–5 years)	237 (21)	130	(22)	107	(20)	
[5–10 years)	249 (22)	131	(22)	118	(22)	
[10–15 years)	143 (13)	69	(12)	74	(14)	
[15–20 years)	97 (9)	45	(8)	52	(10)	
[20–50years)	139 (12)	66	(11)	73	(13)	
> = 50years	40 (4)	24	(4)	16	(3)	
Nb of fever episodes						
1	268	128	(49)	140	(52)	0.25*
2	129	67	(25)	62	(23)	
3	60	28	(11)	32	(12)	
4	45	20	(8)	25	(9)	
5	18	15	(6)	5	(2)	
6 and more	11	5	(2)	6	(2)	
Fever incidence rate (100 pers-year)	77.9	77.8		78.0		0.73♮
Nb of ILI	598	341	(57)	257	(43)	
ILI incidence rate (100 pers-year)	41.3	45.3		36.9		0.09♮
Nb of influenza-positive samples	239	105	(44)	134	(56)	
Influenza incidence rate (100 pers-year)	16.5	13.9		19.2		0.26♮
Nb of malaria-positive tests	178	72	(40)	106	(60)	
Malaria incidence rate (100 pers-year)	12.3	9.6		15.2		0.12♮

* Chi-squared test ^♭^Student's t-test ♮negative binomial regression univariate analysis

A total of 543 inhabitants were identified as febrile during the study period (271 from Dielmo and 272 from Ndiop); 282 (52%) were women and 261 (48%) were men (sex ratio M/F = 0.92 overall and 1.1 in Dielmo and 0.8 in Ndiop, p value = 0.40) (Table 1).

A total of 1,129 episodes of fever were reported: 586 (52%) in Dielmo and 543 (48%) in Ndiop. Patient ages were 1 day to 83.6 years, with a median age of 6.6 years (6.2 years in Dielmo and 6.8 years in Ndiop; p-value = 0.86) (Table 1). The distribution of fever episodes by age group did not differ significantly between the two villages (Table 1).

**Fig 1:**
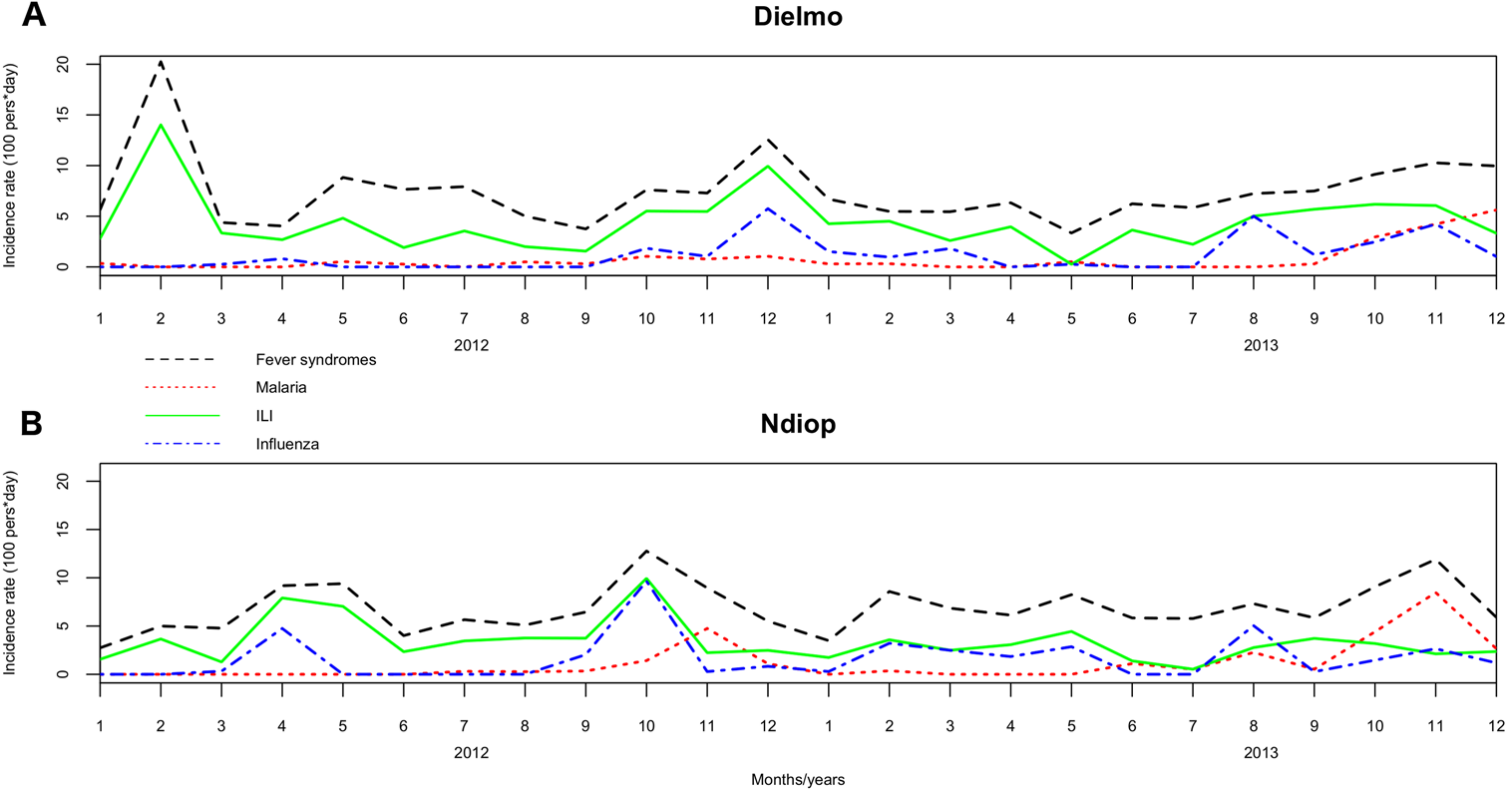
Overall monthly incidence density rates for the two villages, 2012–2013.

**Fig 2:**
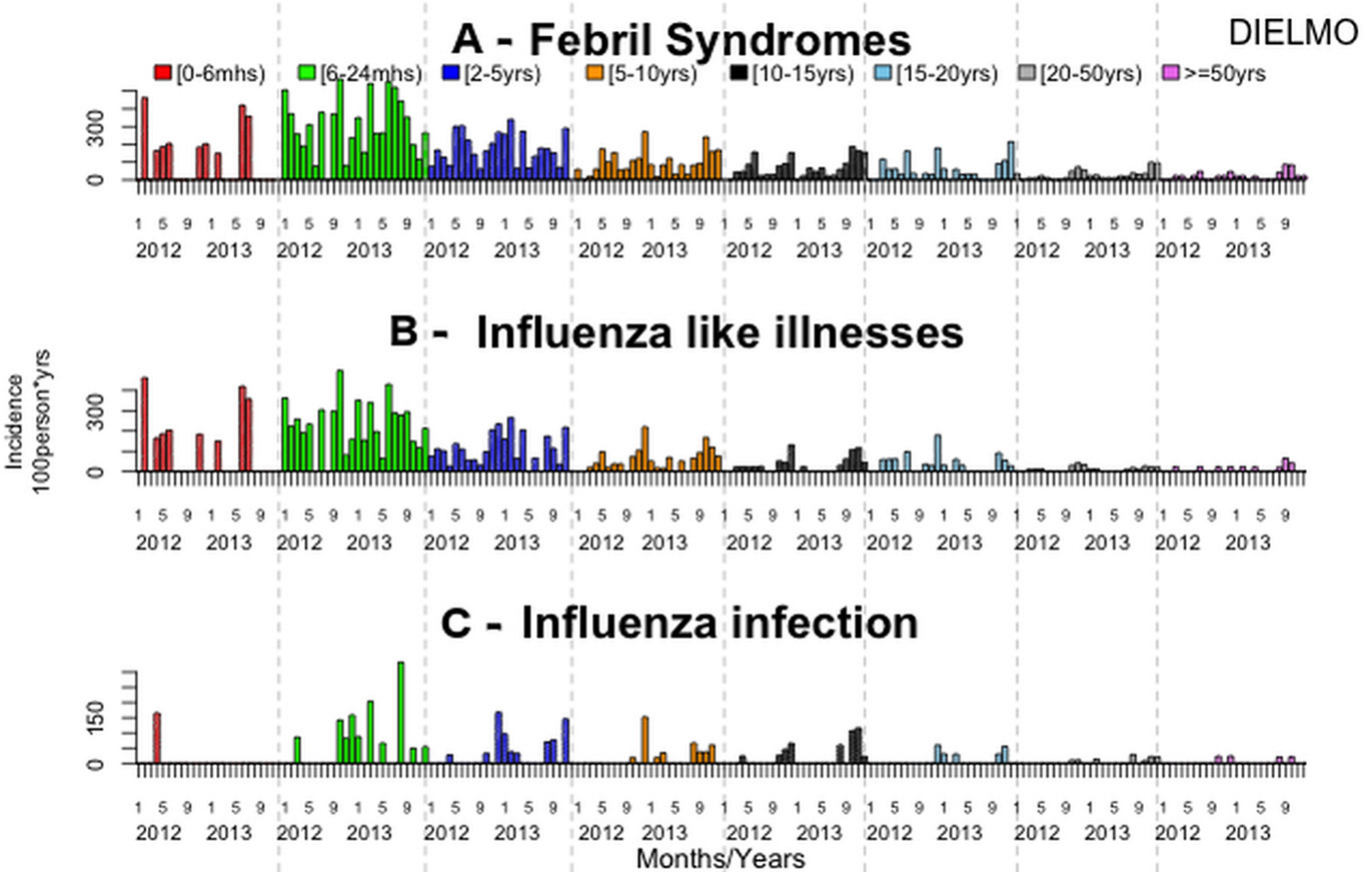
Monthly incidence density rates by age group in Dielmo, 2012–2013.

**Fig 3:**
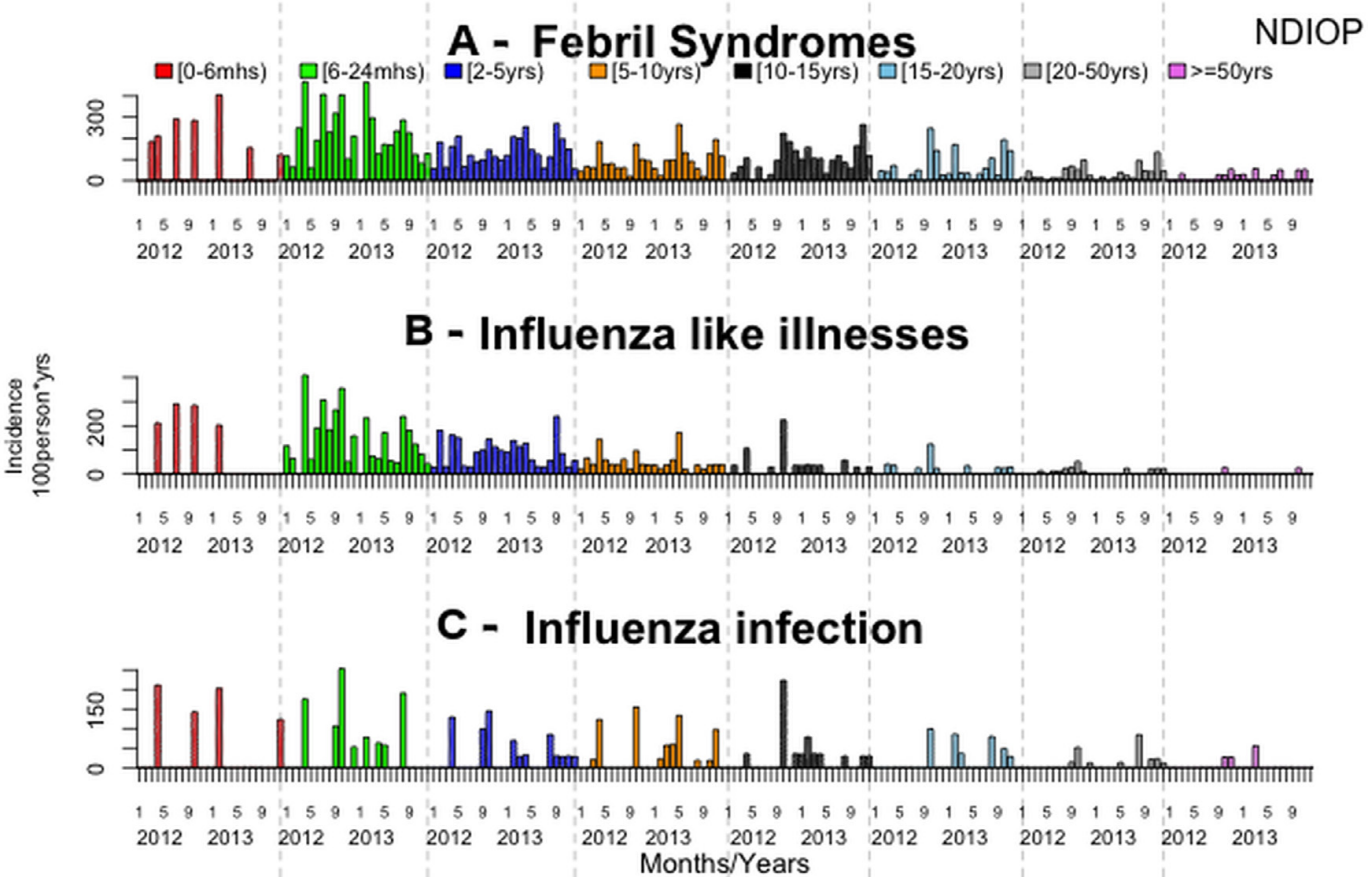
Monthly incidence density rates by age group in Ndiop, 2012–2013.

The overall incidence rate of fever was 77.9 per 100 person-years. The fever incidence rates did not differ significantly between villages (p = 0.54), between years (p = 0.75) or between sexes (p = 0.35). Fever incidence rates differed significantly between age groups, with the [6–24months) and [0–6months) age groups having the highest incidence (Table 2, Figs 1, 2A and 3A, S1 and S2 Tables).

**Table 2:** Incidence of fever syndromes, malaria cases, ILI and influenza infection in Dielmo and Ndiop, January 2012–December 2013.

**DIELMO**	**Fever syndromes**			**Malaria**			**ILI**			**Influenza**		
**2012**	**Crude**	**95%CIs**		**Crude**	**95%CIs**		**Crude**	**95%CIs**		**Crude**	**95%CIs**	
[0–6months)	118.20	117.74	118.66	0.00	0.00	0.00	101.32	100.90	101.74	16.89	16.72	17.06
[6–24months)	287.44	287.00	287.88	0.00	0.00	0.00	222.12	221.73	222.51	39.20	39.04	39.36
[2–5years)	175.25	175.04	175.46	2.40	2.38	2.42	100.83	100.67	100.99	16.81	16.74	16.88
[5–10years)	102.46	102.33	102.59	8.00	7.96	8.04	57.63	57.53	57.73	16.01	15.96	16.06
[10–15years)	63.17	63.06	63.28	7.90	7.86	7.94	29.61	29.53	29.69	13.82	13.77	13.87
[15–20years)	61.63	61.50	61.76	5.36	5.32	5.40	45.55	45.44	45.66	5.36	5.32	5.40
[20–50yrs)	24.22	24.17	24.27	2.79	2.77	2.81	12.11	12.08	12.14	1.86	1.85	1.87
> = 50years	17.80	17.74	17.86	3.96	3.93	3.99	7.91	7.87	7.95	3.96	3.93	3.99
Total	**74.89**	**74.84**	**74.94**	**4.58**	**4.57**	**4.59**	**44.99**	**44.95**	**45.03**	**9.97**	**9.95**	**9.99**
**2013**												
[0–6months)	73.43	73.09	73.77	14.69	14.54	14.84	73.43	73.09	73.77	0.00	0.00	0.00
[6–24months)	334.57	334.14	335.00	0.00	0.00	0.00	236.77	236.41	237.13	66.91	66.72	67.10
[2–5years)	165.00	164.78	165.22	5.79	5.75	5.83	107.10	106.92	107.28	37.63	37.52	37.74
[5–10years)	97.91	97.79	98.03	29.23	29.16	29.30	58.45	58.36	58.54	20.46	20.4	20.52
[10–15years)	74.53	74.40	74.66	16.12	16.06	16.18	30.22	30.14	30.30	24.17	24.1	24.24
[15–20years)	58.92	58.79	59.05	29.46	29.37	29.55	26.78	26.69	26.87	13.39	13.33	13.45
[20–50yrs)	36.16	36.10	36.22	8.14	8.11	8.17	11.75	11.72	11.78	8.14	8.11	8.17
> = 50years	27.25	27.18	27.32	7.27	7.23	7.31	14.54	14.49	14.59	3.63	3.60	3.66
Total	**80.66**	**80.61**	**80.71**	**14.40**	**14.38**	**14.42**	**45.57**	**45.53**	**45.61**	**17.81**	**17.79**	**17.83**
**NDIOP**	**Fever syndromes**			**Malaria**			**ILI**			**Influenza**		
**2012**	**Crude**	**95%CIs**		**Crude**	**95%CIs**		**Crude**	**95%CIs**		**Crude**	**95%CIs**	
[0–6months)	72.10	71.77	72.43	0.00	0.00	0.00	57.68	57.38	57.98	28.84	28.63	29.05
[6–24months)	239.90	239.56	240.24	0.00	0.00	0.00	184.54	184.24	184.84	50.75	50.59	50.91
[2–5years)	116.63	116.45	116.81	0.00	0.00	0.00	95.42	95.26	95.58	29.16	29.07	29.25
[5–10years)	85.76	85.64	85.88	8.41	8.37	8.45	55.49	55.39	55.59	25.22	25.15	25.29
[10–15years)	78.15	78.00	78.30	13.47	13.41	13.53	35.03	34.93	35.13	24.25	24.17	24.33
[15–20years)	61.91	61.78	62.04	33.54	33.44	33.64	23.22	23.14	23.30	10.32	10.27	10.37
[20–50yrs)	33.82	33.76	33.88	5.28	5.26	5.30	12.68	12.64	12.72	6.34	6.31	6.37
> = 50years	14.49	14.43	14.55	2.41	2.39	2.43	2.41	2.39	2.43	4.83	4.79	4.87
Total	**71.95**	**71.90**	**72.00**	**8.59**	**8.57**	**8.61**	**43.82**	**43.78**	**43.86**	**17.77**	**17.75**	**17.79**
**2013**												
[0–6months)	40.69	40.48	40.90	0.00	0.00	0.00	10.17	10.07	10.27	20.34	20.19	20.49
[6–24months)	181.98	181.69	182.27	8.67	8.61	8.73	108.32	108.10	108.54	30.33	30.21	30.45
[2–5years)	155.83	155.63	156.03	19.79	19.72	19.86	86.57	86.42	86.72	27.21	27.13	27.29
[5–10years)	107.11	106.98	107.24	28.77	28.70	28.84	43.16	43.07	43.25	33.57	33.49	33.65
[10–15years)	117.78	117.60	117.96	44.49	44.38	44.60	20.94	20.86	21.02	20.94	20.86	21.02
[15–20years)	70.15	70.01	70.29	30.06	29.97	30.15	10.02	9.97	10.07	22.55	22.47	22.63
[20–50yrs)	41.47	41.40	41.54	17.20	17.16	17.24	8.09	8.06	8.12	14.16	14.12	14.20
> = 50years	22.03	21.96	22.10	6.61	6.57	6.65	2.20	2.18	2.22	4.41	4.38	4.44
Total	**83.73**	**83.68**	**83.78**	**21.49**	**21.46**	**21.52**	**30.42**	**30.39**	**30.45**	**20.65**	**20.63**	**20.67**

Data are cases per 100 person-years

Multivariate analysis including age group, month, year and village as independent variables indicated that fever incidence rates differed significantly between age groups, and between the months from October to December and the rest of the year. The highest risks were in the [6–24month) and [0–6month) age groups (S2 Table).

The most frequently reported associated clinical symptoms were asthenia (83%), headache (67%), cough (52%), and rhinitis (42%) (Table 1). The distribution of the symptoms differed between the villages: asthenia, sweats, chills, anorexia and nausea were significantly more often reported in Ndiop; cough, sputum and rhinitis were more frequently reported in Dielmo (Table 1).

A total of 598 influenza-like illnesses (ILI; 53% of febrile patients) were reported: 341 (57%) in Dielmo and 257 (43%) in Ndiop. The overall ILI incidence rate was 41.3 cases of ILI per 100 person-years, and did not differ significantly between the two villages (p = 0.06), between years (p = 0.15) (Table 1), or between sexes (p = 0.34). The ILI incidence rates differed significantly between age groups, and were highest in the [6–24 month) and [0–6 month) age groups (Table 2, Figs 1, 2B and 3B, S1 and S2 Tables).

Multivariate analysis indicated significant differences in ILI incidence rates according to age group, month (April and from October to December) and village. The risk rates were in the [6–24 month) and [0–6 month) age groups (IRR = 16.1; 95%CI: [11.1–23.3] and IRR = 14.6; 95%CI: [7.9–26.9]) (S2 Table).

Plasmodium carriage was detected in 178 (16%) of the febrile patients: 72 (40%) in Dielmo and 106 (60%) in Ndiop. Among the positive malaria cases, 39 (21%) also presented with ILI symptoms. The malaria incidence rate was 12.3 episodes per 100 person-years during the period, and was not significantly different between the two villages (p = 0.14) or between sexes (p = 0.52). It was significantly higher in 2013 (21.5 cases per 100 person-years in Ndiop; 14.4 in Dielmo). The malaria incidence rate also differed significantly between age groups, being higher in the age groups [10–15 year) and [15–20 year) groups (Table 2, Fig 1, S1 and S2 Tables).

Multivariate analysis indicated significant differences in the malaria incidence rates according to age group, the month (April and from October to December), the year and the village. The highest rates were in the [15–20 year) age group (IRR = 3.3; 95%CI: [2.1–5.3]), in 2013 (IRR = 2.9; 95%CI: [2.0–4.0]), in November (IRR = 23.5; 95%CI: [5.8–96.2]), and in Ndiop (IRR = 1.6; 95%CI: [1.1–2.1]) (S2 Table).

A total of 949 specimens were collected from the 1,129 febrile patients (84%). Influenza A was identified in 158 specimens (17%) and Influenza B in 87 specimens (9%) (Table 3).

**Table 3:** Clinical symptoms associated with influenza infections in Dielmo and Ndiop, January 2012–December 2013.

	Influenza A					Influenza B				
	n	(%)	EF	95% CI	p	n	(%)	EF	95%CI	p
Asthenia	134	(17)	0.29	[-0.43–0.60]	0.27	72	(9)	0.09	[-1.00–0.57]	0.78
Headache	118	(19)	0.60	[0.09–0.83]	0.03	60	(10)	-0.25	[-1.50–0.44]	0.68
Sweat	25	(20)	0.23	[-0.25–0.52]	0.29	4	(3)	-2.33	[-9.00–-0.25]	0.01
Chills	45	(19)	0.17	[-0.25–0.44]	0.36	22	(9)	0.09	[-0.67–0.41]	0.82
Feeling cold	48	(19)	0.09	[-0.25–0.38]	0.60	14	(6)	-1.00	[-2.33–-0.25]	0.01
Thirst	35	(16)	0.00	[-0.67–0.33]	0.92	22	(10)	-0.11	[-0.67–0.44]	0.82
Cough	102	(21)	0.44	[0.23–0.62]	<0.01	69	(14)	0.74	[0.55–0.85]	<0.01
Sputum	28	(34)	0.66	[0.41–0.79]	<0.01	16	(20)	0.63	[0.33–0.79]	<0.01
Dyspnea	10	(23)	0.38	[-0.43–0.69]	0.23	7	(17)	0.52	[-0.25–0.79]	0.10
Sore throat	13	(33)	0.62	[0.23–0.80]	<0.01	5	(13)	0.33	[-1.00–0.74]	0.44
Nasal congestion										
Runny nose	82	(20)	0.33	[0.09–0.52]	0.03	58	(14)	0.64	[0.41–0.78]	<0.01
Stuffy nose	42	(21)	0.29	[-0.11–0.52]	0.06	35	(18)	0.66	[0.44–0.78]	<0.01
Anorexia	62	(16)	-0.11	[-0.67–0.23]	0.49	35	(9)	-0.11	[-0.67–0.33]	0.78
Nausea	15	(12)	-0.67	[-2.33–0.09]	0.10	8	(7)	-0.67	[-2.33–0.29]	0.24
Vomiting	21	(13)	-0.11	[-1.00–0.33]	0.63	6	(4)	-1.50	[-4.00–-0.11]	0.03
Diarrhea	13	(13)	-0.43	[-1.50–0.23]	0.31	7	(7)	-0.43	[-2.33–0.38]	0.41

EF: etiologic fraction

The overall flu incidence density rate was 19.2 per 100 person-years. The flu incidence density rates were not significantly different between villages (p = 0.32), years (p = 0.30) or between sexes (p = 0.78). The flu incidence density rates were significantly different between age groups, the highest being in the [6–24 months) age group (30.3 to 50.7 per 100 person-years) (Table 2, Figs 1, 2C and 3C, S1 and S2 Tables).

Mutivariate analysis revealed significant differences in the flu incidence density rates between age groups, months (April, August and from October to December) and years. They were higher in the [6–24 months) and [0–6 months) age groups (IRR = 7.1; 95%CI: [3.4–4.3] and IRR = 9.9; 95%CI: [2.9–33.6]), in 2013 (IRR = 1.9; 95%CI: [1.3–2.9]), and in October (IRR = 10.3; 95%CI: [3.4–30.9]) (S2 Table).

Cough, sputum and rhinitis were associated with influenza A (RR = 1.8, 2.9 and 1.5, respectively) and B (RR = 3.9, 2.7 and 2.8, respectively) infections; sore throat (RR = 2.6) and headache (RR = 2.5) were associated with Influenza A infections and asthenia with both Influenza A (RR = 1.2) and Influenza B (RR = 1.2) infections (Table 3).

If only ILI had been used as the criterion for sampling, 35% of the influenza A infections 20% of the Influenza B infections would not have been detected (Table 4).

**Table 4:** Distribution of the virus according to ILI definition, village and year, in Dielmo and Ndiop, January 2012–December 2013.

	Influenza A		Influenza B	
	Not ILI	ILI	Not ILI	ILI
**Dielmo**				
2012	2	12	2	21
	(14)	(86)	(9)	(91)
2013	18	37	5	13
	(33)	(67)	(28)	(72)
Total	**20**	**49**	**7**	**34**
	**(29)**	**(71)**	**(17)**	**(83)**
**Ndiop**				
2012	6	35	3	16
	(15)	(85)	(16)	(84)
2013	29	19	7	20
	(60)	(40)	(26)	(74)
**Total**	**35**	**54**	**10**	**36**
	**(39)**	**(61)**	**(22)**	**(78)**
**Total**	**55**	**103**	**17**	**70**
	**(35)**	**(65)**	**(20)**	**(80)**

## Discussion

This is the first study addressing fever syndromes, influenza-like illnesses and influenza infections reported over a substantial period of time by a small cohort established to study malaria. As previously described by Trape *et al*. [23] in Dielmo, the malaria incidence rates in both Dielmo and Ndiop were very low in comparison with the previous periods of the cohort study. Furthermore, we found that malaria rates were highest in the 5–20 year age group. Despite this malaria epidemiological profile, fever syndromes have remained relatively frequent (78 cases per 100 person-years); however, this is largely due to influenza-like illnesses (more than 50% of the cases of fever syndrome). Most such cases were in children under 5 years old. The aims of this study were to investigate the epidemiology of fever syndromes and among them influenza infection in an African rural setting. The motivation was to help reveal the influenza burden in Senegal and thereby to improve the knowledge that can contribute to policy decisions. The most important finding is that the largest proportion of the fever syndromes was not associated with malaria but with ILI and influenza infections. Also, the highest incidence rate of these influenza infections was in the [6–24 month) age group. More than a third of the influenza infections were detected in non-ILI febrile patients. These observations are informative both on issues about influenza surveillance and flu disease burden.

Malaria is a major infectious disease in tropical Africa, and is believed to be the main cause of febrile episodes in children. Not surprisingly, therefore, influenza has received relatively little attention in Africa. The incidence of malaria and prevalence of *P*. *falciparum* have both declined recently in Dielmo [23] and Ndiop [25]. The results we report here are consistent with the trend described by Trape *et al* [23] and the incidence density of malaria attacks being less than 0.5 attacks per person-year in the [10–15 years) age group (the group most often infected).

However, the incidence of fever syndromes remains high from October to December, corresponding to the seasonal malaria transmission period. Here, we report that the spread of influenza was also highest during this period probably in association with the end of the rainy seasons, consistent with observations in Brazil [26]. This type of temporal coincidence between malaria and respiratory infection during the rainy months has also been described elsewhere [27,28].

However, we also found that influenza was present all year round, as in Rwanda [29]. Some studies in Senegal suggest seasonality with influenza peaks in July-August [8], but more recent data from the Senegalese sentinel surveillance network and findings in Gabon [30] are consistent with our results [4]. There is now ample evidence that influenza occurs throughout the year in tropical regions [13].

ILI and influenza incidence density rates were highest among the youngest children, both in Dielmo and Ndiop and in 2012 and 2013, consistent with existing knowledge about respiratory infections in Africa [21]. The highest risks of influenza infection were in [0–6 month) and [6–24 month) age groups, and were 7 to 10-fold higher than in 20 year-old and older age group. Our findings are generally consistent with results from Kenya [3], although the incidence rates we observed are higher than in the study in Kenya.

Our findings about fever syndromes, ILI and influenza infection suggest that prevention strategies should be targeted at children under five and probably younger (less than 24 months old). This would probably reduce the load of respiratory infection in this population and reduce mortality, consistent with the fourth millennium development goal.

Our study has several limitations. First, the diagnostic methods used for influenza virus have limitations: for example, 16% of the cases of fever syndrome were not sampled. These limitations could modify the estimated incidence rates. While we found that influenza was present year round, our study period of 24 months was too short to fully assess whether there are any seasonal patterns.

There may have been information bias concerning the clinical signs, and this may have affected the ILI classification of some fever syndromes and explain the percentage of positive influenza samples not related to ILI.

## Conclusion

In this rural setting in Senegal, the burden of malaria has decreased, but fever, ILI and influenza infection are clearly affecting the population previously affected by malaria: children under 5 years old. If preventive interventions are to be implemented, they certainly have to focus on this population and particularly children under 24 months old.

## Supporting Information

S1 Table Annual incidence rates according to age group and village (episodes per 100 person-months), Dielmo and Ndiop January 2012–December 2013. (DOCX)

S2 Table Results of the univariate and mutivariate binomial negative analyses, for Dielmo and Ndiop, January 2012–December 2013. (DOCX)
