# Supplementary material for: Acute Febrile Illness and Influenza Disease Burden in a Rural Cohort Dedicated to Malaria in Senegal, 2012–2013
Source: PLoS One. 2015 Dec 17;10(12):e0143999. doi: 10.1371/journal.pone.0143999 (PMC4682973; doi:10.1371/journal.pone.0143999)
Supplement: S1 Table — (DOCX) [file pone.0143999.s001.docx]

S1 Table - Annual incidence rates according to age group and village, Dielmo and Ndiop January 2012-December 2013

| **DIELMO** | **[0-6months)** | |  |  | **[6-24months)** | |  |  |
| --- | --- | --- | --- | --- | --- | --- | --- | --- |
| **2012** | Fever synd | Malaria | ILI | Influenza | Fever synd | Malaria | ILI | Influenza |
| **Jan** | 0.00 | 0.00 | 0.00 | 0.00 | 506.29 | 0.00 | 361.63 | 0.00 |
| **Feb** | 462.34 | 0.00 | 462.34 | 0.00 | 371.95 | 0.00 | 223.17 | 0.00 |
| **Mar** | 0.00 | 0.00 | 0.00 | 0.00 | 257.82 | 0.00 | 257.82 | 85.94 |
| **Apr** | 165.27 | 0.00 | 165.27 | 165.27 | 190.73 | 0.00 | 190.73 | 0.00 |
| **May** | 186.35 | 0.00 | 186.35 | 0.00 | 310.85 | 0.00 | 233.14 | 0.00 |
| **Jun** | 204.05 | 0.00 | 204.05 | 0.00 | 77.55 | 0.00 | 0.00 | 0.00 |
| **Jul** | 0.00 | 0.00 | 0.00 | 0.00 | 379.68 | 0.00 | 303.74 | 0.00 |
| **Aug** | 0.00 | 0.00 | 0.00 | 0.00 | 0.00 | 0.00 | 0.00 | 0.00 |
| **Sep** | 0.00 | 0.00 | 0.00 | 0.00 | 371.19 | 0.00 | 296.95 | 0.00 |
| **Oct** | 0.00 | 0.00 | 0.00 | 0.00 | 568.48 | 0.00 | 497.42 | 142.12 |
| **Nov** | 183.54 | 0.00 | 183.54 | 0.00 | 82.08 | 0.00 | 82.08 | 82.08 |
| **Dec** | 202.92 | 0.00 | 0.00 | 0.00 | 237.69 | 0.00 | 158.46 | 158.46 |
| **2013** |  |  |  |  |  |  |  |  |
| **Jan** | 0.00 | 0.00 | 0.00 | 0.00 | 350.36 | 0.00 | 350.36 | 87.59 |
| **Feb** | 149.08 | 149.08 | 149.08 | 0.00 | 155.1 | 0.00 | 155.1 | 0.00 |
| **Mar** | 0.00 | 0.00 | 0.00 | 0.00 | 542.12 | 0.00 | 338.82 | 203.29 |
| **Apr** | 0.00 | 0.00 | 0.00 | 0.00 | 262.3 | 0.00 | 196.72 | 0.00 |
| **May** | 0.00 | 0.00 | 0.00 | 0.00 | 264.67 | 0.00 | 66.17 | 66.17 |
| **Jun** | 419.83 | 0.00 | 419.83 | 0.00 | 552.48 | 0.00 | 429.71 | 0.00 |
| **Jul** | 358.09 | 0.00 | 358.09 | 0.00 | 520.96 | 0.00 | 289.42 | 0.00 |
| **Aug** | 0.00 | 0.00 | 0.00 | 0.00 | 443.4 | 0.00 | 277.12 | 332.55 |
| **Sep** | 0.00 | 0.00 | 0.00 | 0.00 | 352.9 | 0.00 | 294.08 | 0.00 |
| **Oct** | 0.00 | 0.00 | 0.00 | 0.00 | 197.17 | 0.00 | 147.87 | 49.29 |
| **Nov** | 0.00 | 0.00 | 0.00 | 0.00 | 116.88 | 0.00 | 116.88 | 0.00 |
| **Dec** | 0.00 | 0.00 | 0.00 | 0.00 | 265.44 | 0.00 | 212.35 | 53.09 |
| **NDIOP** | **[0-6months)** | |  |  | **[6-24months)** | |  |  |
| **2012** | Fever synd | Malaria | ILI | Influenza | Fever synd | Malaria | ILI | Influenza |
| **Jan** | 0.00 | 0.00 | 0.00 | 0.00 | 115.95 | 0.00 | 115.95 | 0.00 |
| **Feb** | 0.00 | 0.00 | 0.00 | 0.00 | 63.97 | 0.00 | 63.97 | 0.00 |
| **Mar** | 184.47 | 0.00 | 0.00 | 0.00 | 248.47 | 0.00 | 0.00 | 0.00 |
| **Apr** | 211.13 | 0.00 | 211.13 | 211.13 | 467.52 | 0.00 | 409.08 | 175.32 |
| **May** | 0.00 | 0.00 | 0.00 | 0.00 | 60.88 | 0.00 | 60.88 | 0.00 |
| **Jun** | 0.00 | 0.00 | 0.00 | 0.00 | 190.57 | 0.00 | 190.57 | 0.00 |
| **Jul** | 289.88 | 0.00 | 289.88 | 0.00 | 408.1 | 0.00 | 306.08 | 0.00 |
| **Aug** | 0.00 | 0.00 | 0.00 | 0.00 | 228.85 | 0.00 | 183.08 | 0.00 |
| **Sep** | 0.00 | 0.00 | 0.00 | 0.00 | 318.53 | 0.00 | 265.44 | 106.18 |
| **Oct** | 284.24 | 0.00 | 284.24 | 142.12 | 405.27 | 0.00 | 354.61 | 253.29 |
| **Nov** | 0.00 | 0.00 | 0.00 | 0.00 | 102.89 | 0.00 | 51.44 | 0.00 |
| **Dec** | 0.00 | 0.00 | 0.00 | 0.00 | 210.22 | 0.00 | 157.66 | 52.55 |
| **2013** |  |  |  |  |  |  |  |  |
| **Jan** | 0.00 | 0.00 | 0.00 | 0.00 | 0.00 | 0.00 | 0.00 | 0.00 |
| **Feb** | 405.83 | 0.00 | 202.92 | 202.92 | 464.3 | 0.00 | 232.15 | 77.38 |
| **Mar** | 0.00 | 0.00 | 0.00 | 0.00 | 296.35 | 0.00 | 74.09 | 0.00 |
| **Apr** | 0.00 | 0.00 | 0.00 | 0.00 | 126.17 | 0.00 | 63.08 | 63.08 |
| **May** | 0.00 | 0.00 | 0.00 | 0.00 | 171.75 | 0.00 | 171.75 | 57.25 |
| **Jun** | 0.00 | 0.00 | 0.00 | 0.00 | 167.8 | 0.00 | 55.93 | 0.00 |
| **Jul** | 154.77 | 0.00 | 0.00 | 0.00 | 234.74 | 0.00 | 46.95 | 0.00 |
| **Aug** | 0.00 | 0.00 | 0.00 | 0.00 | 286.1 | 0.00 | 238.41 | 190.73 |
| **Sep** | 0.00 | 0.00 | 0.00 | 0.00 | 225.74 | 0.00 | 180.59 | 0.00 |
| **Oct** | 0.00 | 0.00 | 0.00 | 0.00 | 123.4 | 0.00 | 123.4 | 0.00 |
| **Nov** | 0.00 | 0.00 | 0.00 | 0.00 | 82.45 | 41.22 | 82.45 | 0.00 |
| **Dec** | 122.98 | 0.00 | 0.00 | 122.98 | 128.61 | 42.87 | 42.87 | 0.00 |
| Data are cases per 100person-years | | |  |  |  |  |  |  |

| **DIELMO** | **[2-5years)** |  |  |  | **[5-10years)** | |  |  |
| --- | --- | --- | --- | --- | --- | --- | --- | --- |
| **2012** | Fever synd | Malaria | ILI | Influenza | Fever synd | Malaria | ILI | Influenza |
| **Jan** | 76.89 | 0.00 | 76.89 | 0.00 | 59.04 | 19.68 | 0.00 | 0.00 |
| **Feb** | 168.19 | 0.00 | 112.13 | 0.00 | 0.00 | 0.00 | 0.00 | 0.00 |
| **Mar** | 127.71 | 0.00 | 102.17 | 0.00 | 19.97 | 0.00 | 19.97 | 0.00 |
| **Apr** | 80.63 | 0.00 | 26.88 | 26.88 | 60.61 | 0.00 | 40.4 | 0.00 |
| **May** | 300.5 | 27.32 | 136.59 | 0.00 | 173.38 | 0.00 | 96.32 | 0.00 |
| **Jun** | 304.14 | 0.00 | 110.6 | 0.00 | 100.45 | 0.00 | 20.09 | 0.00 |
| **Jul** | 224.6 | 0.00 | 56.15 | 0.00 | 153.39 | 0.00 | 38.35 | 0.00 |
| **Aug** | 144.03 | 0.00 | 57.61 | 0.00 | 56.8 | 0.00 | 37.87 | 0.00 |
| **Sep** | 61.8 | 0.00 | 30.9 | 0.00 | 61.22 | 0.00 | 0.00 | 0.00 |
| **Oct** | 161.76 | 0.00 | 97.05 | 32.35 | 109.36 | 36.45 | 72.9 | 18.23 |
| **Nov** | 205.2 | 0.00 | 205.2 | 0.00 | 121.4 | 17.34 | 104.06 | 0.00 |
| **Dec** | 267.83 | 0.00 | 234.35 | 167.39 | 270.18 | 16.89 | 219.52 | 151.98 |
| **2013** |  |  |  |  |  |  |  |  |
| **Jan** | 256.09 | 0.00 | 160.06 | 96.03 | 85.38 | 0.00 | 51.23 | 0.00 |
| **Feb** | 339.94 | 0.00 | 264.4 | 37.77 | 18.4 | 0.00 | 18.4 | 18.4 |
| **Mar** | 64.7 | 0.00 | 64.7 | 32.35 | 84.47 | 0.00 | 16.89 | 33.79 |
| **Apr** | 273.08 | 0.00 | 204.81 | 0.00 | 121.87 | 0.00 | 69.64 | 0.00 |
| **May** | 66.96 | 0.00 | 0.00 | 0.00 | 31.97 | 15.98 | 0.00 | 0.00 |
| **Jun** | 132.82 | 0.00 | 66.41 | 0.00 | 85.34 | 0.00 | 51.2 | 0.00 |
| **Jul** | 179.22 | 0.00 | 0.00 | 0.00 | 32.03 | 0.00 | 0.00 | 0.00 |
| **Aug** | 173.93 | 0.00 | 173.93 | 69.57 | 82.97 | 0.00 | 66.38 | 66.38 |
| **Sep** | 151.87 | 0.00 | 113.9 | 75.94 | 93.22 | 0.00 | 93.22 | 37.29 |
| **Oct** | 70.78 | 0.00 | 35.39 | 0.00 | 241.52 | 111.47 | 167.2 | 37.16 |
| **Nov** | 290.75 | 72.69 | 218.06 | 145.37 | 159.24 | 119.43 | 119.43 | 59.71 |
| **Dec** | 0.00 | 0.00 | 0.00 | 0.00 | 169.1 | 131.52 | 75.15 | 0.00 |
| **NDIOP** | **[2-5years)** |  |  |  | **[5-10years)** | | |  |
| **2012** | Fever synd | Malaria | ILI | Influenza | Fever synd | Malaria | ILI | Influenza |
| **Jan** | 56.58 | 0.00 | 28.29 | 0.00 | 43.1 | 0.00 | 21.55 | 0.00 |
| **Feb** | 181.42 | 0.00 | 181.42 | 0.00 | 65.85 | 0.00 | 65.85 | 0.00 |
| **Mar** | 62.38 | 0.00 | 31.19 | 0.00 | 60.67 | 0.00 | 40.45 | 20.22 |
| **Apr** | 161.47 | 0.00 | 161.47 | 129.18 | 184.06 | 0.00 | 143.16 | 122.7 |
| **May** | 210.09 | 0.00 | 150.06 | 0.00 | 78.55 | 0.00 | 58.91 | 0.00 |
| **Jun** | 65.93 | 0.00 | 32.96 | 0.00 | 80.76 | 0.00 | 40.38 | 0.00 |
| **Jul** | 119.95 | 0.00 | 29.99 | 0.00 | 59.49 | 0.00 | 39.66 | 0.00 |
| **Aug** | 89.52 | 0.00 | 89.52 | 0.00 | 60.88 | 0.00 | 60.88 | 0.00 |
| **Sep** | 98.54 | 0.00 | 98.54 | 98.54 | 20.37 | 0.00 | 20.37 | 0.00 |
| **Oct** | 144.8 | 0.00 | 144.8 | 144.8 | 173.56 | 19.28 | 96.42 | 154.28 |
| **Nov** | 113.55 | 0.00 | 113.55 | 0.00 | 100.79 | 40.31 | 40.31 | 0.00 |
| **Dec** | 97.31 | 0.00 | 97.31 | 0.00 | 93.13 | 37.25 | 37.25 | 0.00 |
| **2013** |  |  |  |  |  |  |  |  |
| **Jan** | 119.66 | 0.00 | 89.74 | 0.00 | 56.37 | 0.00 | 37.58 | 0.00 |
| **Feb** | 207.14 | 0.00 | 138.09 | 69.05 | 22.45 | 0.00 | 22.45 | 22.45 |
| **Mar** | 199.12 | 0.00 | 113.79 | 28.45 | 95.27 | 0.00 | 38.11 | 57.16 |
| **Apr** | 253.65 | 0.00 | 126.82 | 31.71 | 98.19 | 0.00 | 58.91 | 58.91 |
| **May** | 145.52 | 0.00 | 58.21 | 0.00 | 265.91 | 0.00 | 170.94 | 132.96 |
| **Jun** | 123.08 | 30.77 | 30.77 | 0.00 | 132.2 | 18.89 | 18.89 | 0.00 |
| **Jul** | 59.44 | 0.00 | 29.72 | 0.00 | 91.73 | 18.35 | 0.00 | 0.00 |
| **Aug** | 112.56 | 0.00 | 56.28 | 84.42 | 55.23 | 18.41 | 36.82 | 18.41 |
| **Sep** | 268.57 | 0.00 | 238.73 | 29.84 | 19.51 | 0.00 | 19.51 | 0.00 |
| **Oct** | 196.37 | 84.16 | 84.16 | 28.05 | 126.45 | 90.32 | 36.13 | 18.06 |
| **Nov** | 148.23 | 118.59 | 29.65 | 29.65 | 194.08 | 135.85 | 38.82 | 97.04 |
| **Dec** | 55.13 | 0.00 | 55.13 | 27.57 | 115.83 | 57.91 | 38.61 | 0.00 |
| Data are cases per 100person-years | | |  |  |  |  |  |  |

| **DIELMO** | **[10-15years)** | |  |  | **[15-20years)** | |  |  |
| --- | --- | --- | --- | --- | --- | --- | --- | --- |
| **2012** | Fever synd | Malaria | ILI | Influenza | Fever synd | Malaria | ILI | Influenza |
| **Jan** | 0.00 | 0.00 | 0.00 | 0.00 | 0.00 | 0.00 | 0.00 | 0.00 |
| **Feb** | 0.00 | 0.00 | 0.00 | 0.00 | 0.00 | 0.00 | 0.00 | 0.00 |
| **Mar** | 43.64 | 0.00 | 21.82 | 0.00 | 116.79 | 0.00 | 58.39 | 0.00 |
| **Apr** | 45.94 | 0.00 | 22.97 | 22.97 | 60.57 | 0.00 | 60.57 | 0.00 |
| **May** | 87.28 | 21.82 | 21.82 | 0.00 | 61.85 | 0.00 | 61.85 | 0.00 |
| **Jun** | 156.95 | 22.42 | 22.42 | 0.00 | 31.82 | 0.00 | 0.00 | 0.00 |
| **Jul** | 24.58 | 0.00 | 24.58 | 0.00 | 161.47 | 0.00 | 96.88 | 0.00 |
| **Aug** | 28.74 | 0.00 | 0.00 | 0.00 | 36.97 | 36.97 | 0.00 | 0.00 |
| **Sep** | 29.17 | 0.00 | 0.00 | 0.00 | 0.00 | 0.00 | 0.00 | 0.00 |
| **Oct** | 77.22 | 25.74 | 51.48 | 25.74 | 36.06 | 0.00 | 36.06 | 0.00 |
| **Nov** | 90.52 | 0.00 | 45.26 | 45.26 | 29.62 | 0.00 | 29.62 | 0.00 |
| **Dec** | 152.92 | 21.85 | 131.07 | 65.54 | 178.75 | 29.79 | 178.75 | 59.58 |
| **2013** |  |  |  |  |  |  |  |  |
| **Jan** | 0.00 | 0.00 | 0.00 | 0.00 | 61.8 | 30.9 | 30.9 | 30.9 |
| **Feb** | 24.16 | 0.00 | 24.16 | 0.00 | 0.00 | 0.00 | 0.00 | 0.00 |
| **Mar** | 66.05 | 0.00 | 0.00 | 0.00 | 59.54 | 0.00 | 59.54 | 29.77 |
| **Apr** | 45.77 | 0.00 | 0.00 | 0.00 | 30.29 | 0.00 | 30.29 | 0.00 |
| **May** | 66.61 | 22.2 | 0.00 | 0.00 | 31.9 | 0.00 | 0.00 | 0.00 |
| **Jun** | 22.7 | 0.00 | 0.00 | 0.00 | 32.01 | 0.00 | 0.00 | 0.00 |
| **Jul** | 25.83 | 0.00 | 0.00 | 0.00 | 0.00 | 0.00 | 0.00 | 0.00 |
| **Aug** | 59.34 | 0.00 | 29.67 | 59.34 | 0.00 | 0.00 | 0.00 | 0.00 |
| **Sep** | 93.1 | 31.03 | 62.06 | 0.00 | 0.00 | 0.00 | 0.00 | 0.00 |
| **Oct** | 186.49 | 26.64 | 106.56 | 106.56 | 91.46 | 60.98 | 91.46 | 30.49 |
| **Nov** | 162.54 | 69.66 | 116.1 | 116.1 | 110.1 | 27.52 | 55.05 | 55.05 |
| **Dec** | 156.38 | 44.68 | 44.68 | 22.34 | 214.85 | 188 | 26.86 | 0.00 |
| **NDIOP** | **[10-15years)** | |  |  | **[15-20years)** | |  |  |
| **2012** | Fever synd | Malaria | ILI | Influenza | Fever synd | Malaria | ILI | Influenza |
| **Jan** | 0.00 | 0.00 | 0.00 | 0.00 | 0.00 | 0.00 | 0.00 | 0.00 |
| **Feb** | 35.95 | 0.00 | 35.95 | 0.00 | 45.54 | 0.00 | 0.00 | 0.00 |
| **Mar** | 65.17 | 0.00 | 0.00 | 0.00 | 40.49 | 0.00 | 40.49 | 0.00 |
| **Apr** | 105.87 | 0.00 | 105.87 | 35.29 | 71.2 | 0.00 | 35.6 | 0.00 |
| **May** | 0.00 | 0.00 | 0.00 | 0.00 | 0.00 | 0.00 | 0.00 | 0.00 |
| **Jun** | 62.54 | 0.00 | 0.00 | 0.00 | 0.00 | 0.00 | 0.00 | 0.00 |
| **Jul** | 0.00 | 0.00 | 0.00 | 0.00 | 28.87 | 28.87 | 0.00 | 0.00 |
| **Aug** | 28.78 | 0.00 | 28.78 | 0.00 | 49.56 | 24.78 | 24.78 | 0.00 |
| **Sep** | 95.37 | 0.00 | 0.00 | 0.00 | 0.00 | 0.00 | 0.00 | 0.00 |
| **Oct** | 223.49 | 0.00 | 223.49 | 223.49 | 247.12 | 98.85 | 123.56 | 98.85 |
| **Nov** | 184.31 | 122.88 | 0.00 | 0.00 | 142.49 | 142.49 | 23.75 | 0.00 |
| **Dec** | 142.26 | 35.56 | 35.56 | 35.56 | 25.15 | 25.15 | 0.00 | 0.00 |
| **2013** |  |  |  |  |  |  |  |  |
| **Jan** | 102.03 | 0.00 | 34.01 | 34.01 | 31.35 | 0.00 | 0.00 | 0.00 |
| **Feb** | 156.59 | 39.15 | 39.15 | 78.3 | 170.48 | 0.00 | 0.00 | 85.24 |
| **Mar** | 105.46 | 0.00 | 35.15 | 35.15 | 36.02 | 0.00 | 0.00 | 36.02 |
| **Apr** | 103.37 | 0.00 | 34.46 | 34.46 | 33.69 | 0.00 | 33.69 | 0.00 |
| **May** | 32.58 | 0.00 | 0.00 | 0.00 | 0.00 | 0.00 | 0.00 | 0.00 |
| **Jun** | 94.71 | 31.57 | 0.00 | 0.00 | 31.27 | 0.00 | 0.00 | 0.00 |
| **Jul** | 117.63 | 0.00 | 0.00 | 0.00 | 57.47 | 28.74 | 0.00 | 0.00 |
| **Aug** | 85.94 | 57.29 | 57.29 | 28.65 | 105.64 | 79.23 | 0.00 | 79.23 |
| **Sep** | 59.29 | 29.65 | 0.00 | 0.00 | 26.43 | 0.00 | 26.43 | 0.00 |
| **Oct** | 164.4 | 82.2 | 27.4 | 0.00 | 192.62 | 96.31 | 24.08 | 48.15 |
| **Nov** | 264.67 | 176.45 | 0.00 | 29.41 | 140.81 | 112.64 | 28.16 | 28.16 |
| **Dec** | 117.16 | 87.87 | 29.29 | 29.29 | 0.00 | 0.00 | 0.00 | 0.00 |
| Data are cases per 100person-years | | |  |  |  |  |  |  |

| **DIELMO** | **[20-50yrs)** | |  |  | **>=50years** | | |  | |  |
| --- | --- | --- | --- | --- | --- | --- | --- | --- | --- | --- |
| **2012** | Fever synd | Malaria | ILI | Influenza | Fever synd | Malaria | | ILI | | Influenza |
| **Jan** | 34.87 | 0.00 | 0.00 | 0.00 | 0.00 | 0.00 | | 0.00 | | 0.00 |
| **Feb** | 0.00 | 0.00 | 0.00 | 0.00 | 0.00 | 0.00 | | 0.00 | | 0.00 |
| **Mar** | 10.63 | 0.00 | 10.63 | 0.00 | 23.58 | 0.00 | | 23.58 | | 0.00 |
| **Apr** | 11.54 | 0.00 | 11.54 | 0.00 | 23.58 | 0.00 | | 0.00 | | 0.00 |
| **May** | 23.85 | 0.00 | 11.92 | 0.00 | 0.00 | 0.00 | | 0.00 | | 0.00 |
| **Jun** | 12.05 | 0.00 | 0.00 | 0.00 | 23.84 | 0.00 | | 0.00 | | 0.00 |
| **Jul** | 0.00 | 0.00 | 0.00 | 0.00 | 47.78 | 0.00 | | 23.89 | | 0.00 |
| **Aug** | 0.00 | 0.00 | 0.00 | 0.00 | 0.00 | 0.00 | | 0.00 | | 0.00 |
| **Sep** | 11.14 | 11.14 | 0.00 | 0.00 | 0.00 | 0.00 | | 0.00 | | 0.00 |
| **Oct** | 49.45 | 9.89 | 29.67 | 9.89 | 22.74 | 0.00 | | 22.74 | | 22.74 |
| **Nov** | 73.55 | 10.51 | 42.03 | 10.51 | 23.72 | 23.72 | | 0.00 | | 0.00 |
| **Dec** | 55.63 | 0.00 | 33.38 | 0.00 | 47.25 | 23.63 | | 23.63 | | 23.63 |
| **2013** |  |  |  |  |  |  | |  | |  |
| **Jan** | 23.29 | 0.00 | 11.64 | 0.00 | 21.83 | 0.00 | | 0.00 | | 0.00 |
| **Feb** | 26.87 | 0.00 | 13.43 | 13.43 | 23.75 | 0.00 | | 23.75 | | 0.00 |
| **Mar** | 11.91 | 0.00 | 0.00 | 0.00 | 0.00 | 0.00 | | 0.00 | | 0.00 |
| **Apr** | 11.78 | 0.00 | 0.00 | 0.00 | 21.63 | 0.00 | | 21.63 | | 0.00 |
| **May** | 11.54 | 0.00 | 0.00 | 0.00 | 0.00 | 0.00 | | 0.00 | | 0.00 |
| **Jun** | 22.16 | 0.00 | 0.00 | 0.00 | 0.00 | 0.00 | | 0.00 | | 0.00 |
| **Jul** | 20.05 | 0.00 | 10.03 | 0.00 | 0.00 | 0.00 | | 0.00 | | 0.00 |
| **Aug** | 38.12 | 0.00 | 19.06 | 28.59 | 41.13 | 0.00 | | 20.57 | | 20.57 |
| **Sep** | 30.5 | 0.00 | 10.17 | 0.00 | 86.86 | 0.00 | | 65.15 | | 0.00 |
| **Oct** | 36.13 | 9.03 | 27.1 | 9.03 | 83.58 | 41.79 | | 41.79 | | 20.9 |
| **Nov** | 98.21 | 32.74 | 21.83 | 21.83 | 22.33 | 22.33 | | 0.00 | | 0.00 |
| **Dec** | 94.95 | 52.75 | 21.1 | 21.1 | 22.9 | 22.9 | | 0.00 | | 0.00 |
| **NDIOP** | **[20-50yrs)** | |  |  | **>=50years** | | |  | |  |
| **2012** | Fever synd | Malaria | ILI | Influenza | Fever synd | | Malaria | | ILI | Influenza |
| **Jan** | 13.2 | 0.00 | 0.00 | 0.00 | 0.00 | | 0.00 | | 0.00 | 0.00 |
| **Feb** | 42.75 | 0.00 | 0.00 | 0.00 | 0.00 | | 0.00 | | 0.00 | 0.00 |
| **Mar** | 14.21 | 0.00 | 0.00 | 0.00 | 30.95 | | 0.00 | | 0.00 | 0.00 |
| **Apr** | 13.43 | 0.00 | 13.43 | 0.00 | 0.00 | | 0.00 | | 0.00 | 0.00 |
| **May** | 0.00 | 0.00 | 0.00 | 0.00 | 0.00 | | 0.00 | | 0.00 | 0.00 |
| **Jun** | 12.16 | 0.00 | 12.16 | 0.00 | 0.00 | | 0.00 | | 0.00 | 0.00 |
| **Jul** | 11.49 | 0.00 | 11.49 | 0.00 | 0.00 | | 0.00 | | 0.00 | 0.00 |
| **Aug** | 57.63 | 0.00 | 23.05 | 0.00 | 0.00 | | 0.00 | | 0.00 | 0.00 |
| **Sep** | 68.22 | 13.64 | 27.29 | 13.64 | 27.24 | | 0.00 | | 0.00 | 0.00 |
| **Oct** | 51.55 | 0.00 | 51.55 | 51.55 | 26.62 | | 0.00 | | 26.62 | 26.62 |
| **Nov** | 96.6 | 48.3 | 12.07 | 0.00 | 53.48 | | 26.74 | | 0.00 | 26.74 |
| **Dec** | 23.38 | 0.00 | 0.00 | 11.69 | 26.58 | | 0.00 | | 0.00 | 0.00 |
| **2013** |  |  |  |  |  | |  | |  |  |
| **Jan** | 0.00 | 0.00 | 0.00 | 0.00 | 28.42 | | 0.00 | | 0.00 | 0.00 |
| **Feb** | 16.98 | 0.00 | 0.00 | 0.00 | 0.00 | | 0.00 | | 0.00 | 0.00 |
| **Mar** | 0.00 | 0.00 | 0.00 | 0.00 | 55.55 | | 0.00 | | 0.00 | 55.55 |
| **Apr** | 13.92 | 0.00 | 0.00 | 0.00 | 0.00 | | 0.00 | | 0.00 | 0.00 |
| **May** | 38.09 | 0.00 | 0.00 | 12.7 | 0.00 | | 0.00 | | 0.00 | 0.00 |
| **Jun** | 23.44 | 11.72 | 23.44 | 0.00 | 26.16 | | 0.00 | | 0.00 | 0.00 |
| **Jul** | 10.74 | 0.00 | 0.00 | 0.00 | 51.08 | | 0.00 | | 0.00 | 0.00 |
| **Aug** | 94.27 | 31.42 | 0.00 | 83.8 | 0.00 | | 0.00 | | 0.00 | 0.00 |
| **Sep** | 46.48 | 11.62 | 0.00 | 0.00 | 0.00 | | 0.00 | | 0.00 | 0.00 |
| **Oct** | 41.91 | 20.96 | 20.96 | 20.96 | 49.36 | | 24.68 | | 24.68 | 0.00 |
| **Nov** | 134.08 | 89.39 | 22.35 | 22.35 | 51.26 | | 51.26 | | 0.00 | 0.00 |
| **Dec** | 44.87 | 22.44 | 22.44 | 11.22 | 0.00 | | 0.00 | | 0.00 | 0.00 |
| Data are cases per 100person-years | | |  |  |  | |  | |  |  |
