# Supplementary material for: Acute Febrile Illness and Influenza Disease Burden in a Rural Cohort Dedicated to Malaria in Senegal, 2012–2013
Source: PLoS One. 2015 Dec 17;10(12):e0143999. doi: 10.1371/journal.pone.0143999 (PMC4682973; doi:10.1371/journal.pone.0143999)
Supplement: S2 Table — (DOCX) [file pone.0143999.s002.docx]

Supplemental file 2 - Results of the univariate and mutivariate binomial negative analyses, for Dielmo and Ndiop, January 2012-December 2013

|  | Crude IRR | 95%CI | p-value | IRR adjusted | 95% CI | p-value |
| --- | --- | --- | --- | --- | --- | --- |
| **Fever incidence rates** | | | | | | |
| Village |  |  |  |  |  |  |
| Ndiop | 0.9 | [0.7-1.2] | 0.54 | 0.9 | [0.8-1.1] | 0.34 |
| Dielmo | 1.0 |  |  |  |  |  |
| Years |  |  |  |  |  |  |
| 2012 | 1.0 |  |  |  |  |  |
| 2013 | 1.1 | [0.8-1.3] | 0.75 | 1.1 | [0.9-1.3] | 0.15 |
| Months |  |  |  |  |  |  |
| Jan | 1.0 |  |  |  |  |  |
| Feb | 1.5 | [0.8-2.7] | 0.17 | 1.2 | [0.8-1.8] | 0.31 |
| Mar | 1.1 | [0.6-1.9] | 0.71 | 1.0 | [0.7-1.5] | 0.89 |
| Apr | 1.3 | [0.8-2.3] | 0.29 | 1.2 | [0.9-1.7] | 0.43 |
| May | 1.5 | [0.8-2.6] | 0.19 | 1.2 | [0.8-1.8] | 0.26 |
| Jun | 1.3 | [0.7-2.2] | 0.38 | 1.1 | [0.8-1.6] | 0.56 |
| Jul | 1.4 | [0.8-2.5] | 0.22 | 1.2 | [0.8-1.8] | 0.31 |
| Aug | 1.2 | [0.7-2.1] | 0.52 | 1.2 | [0.8-1.8] | 0.37 |
| Sep | 1.2 | [0.7-2.1] | 0.55 | 1.1 | [0.8-1.7] | 0.50 |
| Oct | 2.1 | [1.2-3.6] | <0.01 | 1.8 | [1.3-2.6] | <0.01 |
| Nov | 1.9 | [1.1-3.3] | 0.02 | 1.8 | [1.3-2.6] | <0.01 |
| Dec | 1.8 | [1.1-3.2] | 0.03 | 1.6 | [1.1-2.4] | <0.01 |
| Age Groups |  |  |  |  |  |  |
| >=50years | 0.9 | [0.6-1.4] | 0.90 | 0.9 | [0.8-1.1] |  |
| [20-50 years) | 1.0 |  |  | 1.0 |  |  |
| [15-20 years) | 2.2 | [1.6-2.9] | <0.01 | 2.2 | [1.6-2.9] | <0.01 |
| [10-15 years) | 2.5 | [1.9-3.2] | <0.01 | 2.5 | [1.9-3.2] | <0.01 |
| [5-10 years) | 2.6 | [2.0-3.4] | <0.01 | 2.7 | [2.1-3.4] | <0.01 |
| [2-5 years) | 4.1 | [3.2-5.3] | <0.01 | 4.4 | [3.4-5.5] | <0.01 |
| [6-24months) | 7.0 | [5.4-9.1] | <0.01 | 7.3 | [5.7-9.3] | <0.01 |
| [0-6months) | 6.3 | [3.8-10.3] | <0.01 | 6.4 | [3.9-10.4] | <0.01 |

|  | **Crude IRR** | **95%CI** | **p-value** | **IRR adjusted** | **95% CI** | **p-value** |
| --- | --- | --- | --- | --- | --- | --- |
| **ILI incidence rates** | | | | | | |
| Village |  |  |  |  |  |  |
| Ndiop | 0.7 | [0.5-1.0] | 0.06 | 0.7 | [0.6-0.9] | <0.01 |
| Dielmo | 1.0 |  |  | 1.0 |  |  |
| Years |  |  |  |  |  |  |
| 2012 | 1.0 |  |  | 1.0 |  |  |
| 2013 | 0.8 | [0.6-1.1] | 0.15 | 0.8 | [0.7-1.0] | 0.09 |
| Months |  |  |  |  |  |  |
| Jan | 1.0 |  | 0.21 | 1.0 |  |  |
| Feb | 1.6 | [0.7-3.6] | 0.29 | 1.4 | [0.8-2.3] | 0.19 |
| Mar | 0.9 | [0.4-1.9] | 0.75 | 0.9 | [0.5-1.5] | 0.67 |
| Apr | 1.5 | [0.7-3.4] | 0.28 | 1.5 | [0.9-2.5] | 0.94 |
| May | 1.2 | [0.5-2.8] | 0.64 | 1.1 | [0.7-1.9] | 0.65 |
| Jun | 0.8 | [0.4-1.8] | 0.61 | 0.8 | [0.5-1.4] | 0.45 |
| Jul | 0.9 | [0.4-1.9] | 0.70 | 0.8 | [0.5-1.4] | 0.54 |
| Aug | 1.2 | [0.5-2.7] | 0.67 | 1.3 | [0.8-2.1] | 0.33 |
| Sep | 1.3 | [0.6-2.9] | 0.51 | 1.3 | [0.8-2.2] | 0.24 |
| Oct | 2.2 | [1.0-4.7] | 0.04 | 2.3 | [1.5-3.7] | <0.01 |
| Nov | 1.4 | [0.6-3.1] | 0.39 | 1.4 | [0.9-2.3] | 0.14 |
| Dec | 1.6 | [0.7-3.6] | 0.22 | 1.7 | [1.1-2.7] | 0.03 |
| Age Groups |  |  |  |  |  |  |
| >=50years | 1.0 | [0.5-1.9] | 0.92 | 0.9 | [0.5-1.7] | 0.87 |
| [20-50 years) | 1.0 |  |  | 1.0 |  |  |
| [15-20 years) | 2.7 | [1.7-4.4] | <0.01 | 2.7 | [1.7-4.3] | <0.01 |
| [10-15 years) | 2.6 | [1.7-4.1] | <0.01 | 2.6 | [1.7-4.0] | <0.01 |
| [5-10 years) | 4.3 | [2.9-6.4] | <0.01 | 4.5 | [3.1-6.5] | <0.01 |
| [2-5 years) | 7.9 | [5.4-11.6] | <0.01 | 8.4 | [5.9-12.2] | <0.01 |
| [6-24months) | 15.2 | [10.3-22.3] | <0.01 | 16.1 | [11.1-23.3] | <0.01 |
| [0-6months) | 14.6 | [7.8-27.3] | <0.01 | 14.6 | [7.9-26.9] | <0.01 |
|  |  |  |  |  |  |  |

|  |  |  |  |  |  |  |
| --- | --- | --- | --- | --- | --- | --- |
|  | **Crude IRR** | **95%CI** | **p-value** | **IRR adjusted** | **95% CI** | **p-value** |
| **Malaria incidence rates** | | | | | | |
| Village |  |  |  |  |  |  |
| Ndiop | 1.5 | [0.9-2.7] | 0.14 | 1.6 | [1.2-2.1] | <0.01 |
| Dielmo | 1.0 |  |  |  |  |  |
| Years |  |  |  |  |  |  |
| 2012 | 1.0 |  |  |  |  |  |
| 2013 | 2.6 | [1.4-4.6] | <0.01 | 2.9 | [2.0-4.0] | <0.01 |
| Months |  |  |  |  |  |  |
| Jan | 1.0 |  |  |  |  |  |
| Feb | 1.2 | [0.2-8.7] | 0.86 | 1.1 | [0.1-7.4] | 0.96 |
| Mar | 0.0 | -- | 0.99 | 0.0 | -- | 0.99 |
| Apr | 0.0 | -- | 0.99 | 0.0 | -- | 0.99 |
| May | 2.0 | [0.4-1.1] | 0.43 | 1.9 | [0.3-10.5] | 0.44 |
| Jun | 2.1 | [0.4-1.2] | 0.37 | 1.9 | [0.4-9.8] | 0.43 |
| Jul | 1.3 | [0.2-8.2] | 0.75 | 1.2 | [0.2-7.0] | 0.85 |
| Aug | 5.1 | [1.1-2.4] | 0.04 | 4.3 | [1.0-1.9] | 0.06 |
| Sep | 2.3 | [0.4-1.3] | 0.33 | 2.1 | [0.4-11.0] | 0.36 |
| Oct | 12.9 | [3.0-55.7] | <0.01 | 13.0 | [3.1-53.9] | <0.01 |
| Nov | 27.5 | [5.6-99.6] | <0.01 | 23.5 | [5.8-96.2] | <0.01 |
| Dec | 14.5 | [3.3-62.5] | <0.01 | 15.5 | [3.7-64.4] | <0.01 |
| Age Groups |  |  |  |  |  |  |
| >=50years | 1.0 | [0.4-2.8] | 0.97 | 0.8 | [0.4-1.7] | 0.60 |
| [20-50 years) | 1.0 |  |  | 1.0 |  |  |
| [15-20 years) | 3.3 | [1.4-7.6] | <0.01 | 3.3 | [2.1-5.3] | <0.01 |
| [10-15 years) | 2.5 | [1.1-5.6] | 0.02 | 2.5 | [1.5-4.0] | <0.01 |
| [5-10 years) | 2.1 | [0.9-4.6] | 0.06 | 2.4 | [1.5-3.7] | <0.01 |
| [2-5 years) | 0.8 | [0.3-2.0] | 0.62 | 0.9 | [0.5-1.9] | 0.94 |
| [6-24months) | 0.3 | [0.1-1.3] | 0.10 | 0.3 | [0.1-1.1] | 0.06 |
| [0-6months) | 1.2 | [0.1-1.1] | 0.86 | 0.2 | [0.2-1.2] | 0.64 |

|  | **Crude IRR** | **95%CI** | **p-value** | **IRR adjusted** | **95% CI** | **p-value** |
| --- | --- | --- | --- | --- | --- | --- |
| **Flu incidence rates** | | | | | | |
| Village |  |  |  |  |  |  |
| Ndiop | 1.3 | [0.8-2.1] | 0.32 | 1.4 | [0.9-2.0] | 0.10 |
| Dielmo | 1.0 |  |  |  |  |  |
| Years |  |  |  |  |  |  |
| 2012 | 1.0 |  |  |  |  |  |
| 2013 | 1.3 | [08-2.1] | 0.31 | 1.8 | [1.2-2.6] | <0.01 |
| Months |  |  |  |  |  |  |
| Jan | 1.0 |  |  |  |  |  |
| Feb | 2.4 | [0.7-8.8] | 0.18 | 1.8 | [0.5-5.7] | 0.33 |
| Mar | 2.3 | [0.7-7.9] | 0.18 | 2.1 | [0.7-6.5] | 0.18 |
| Apr | 4.4 | [1.3-14.4] | 0.01 | 3.0 | [1.0-8.6] | 0.04 |
| May | 1.5 | [0.4-5.5] | 0.57 | 1.2 | [0.3-4.0] | 0.79 |
| Jun | 0.0 |  | 0.99 | 0.0 |  | 0.99 |
| Jul | 0.0 |  | 0.99 | 0.0 |  | 0.99 |
| Aug | 4.3 | [1.3-14.3] | 0.02 | 4.6 | [1.6-13.2] | <0.01 |
| Sep | 1.8 | [0.5-6.6] | 0.36 | 1.5 | [0.4-4.7] | 0.53 |
| Oct | 10.9 | [3.2-31.9] | <0.01 | 7.5 | [2.7-20.9] | <0.01 |
| Nov | 3.9 | [1.2-13.0] | 0.02 | 3.5 | [1.2-10.1] | 0.02 |
| Dec | 5.9 | [1.8-19.1] | <0.01 | 4.7 | [1.6-13.4] | <0.01 |
| Age Groups |  |  |  |  |  |  |
| >=50years | 0.9 | [0.3-2.5] | 0.83 | 0.9 | [0.3-2.4] | 0.82 |
| [20-50 years) | 1.0 |  |  |  |  |  |
| [15-20 years) | 2.0 | [0.9-4.7] | 0.10 | 2.0 | [0.9-4.5] | 0.07 |
| [10-15 years) | 2.9 | [1.3-6.2] | <0.01 | 3.0 | [1.5-6.1] | <0.01 |
| [5-10 years) | 2.9 | [1.4-5.9] | <0.01 | 3.6 | [1.8-6.9] | <0.01 |
| [2-5 years) | 3.5 | [1.6-7.3] | <0.01 | 4.6 | [2.3-9.1] | <0.01 |
| [6-24months) | 5.8 | [2.7-12.4] | <0.01 | 7.1 | [3.6-14.3] | <0.01 |
| [0-6months) | 6.6 | [1.9-23.1] | <0.01 | 9.9 | [2.9-33.6] | <0.01 |
